# Combination of Matching Responsive Stimulations of Hippocampus and Subiculum for Effective Seizure Suppression in Temporal Lobe Epilepsy

**DOI:** 10.3389/fneur.2021.638795

**Published:** 2021-08-26

**Authors:** Fang Zhang, Yufang Yang, Yongte Zheng, Junming Zhu, Ping Wang, Kedi Xu

**Affiliations:** ^1^Qiushi Academy for Advanced Studies, Zhejiang University, Hangzhou, China; ^2^Key Laboratory of Biomedical Engineering of Education Ministry, Department of Biomedical Engineering Zhejiang University, Hangzhou, China; ^3^Zhejiang Provincial Key Laboratory of Cardio-Cerebral Vascular Detection Technology and Medicinal Effectiveness Appraisal, Zhejiang University, Hangzhou, China; ^4^Department of Neurosurgery, Second Affiliated Hospital of Zhejiang University, Hangzhou, China

**Keywords:** combined stimulation, granger causality, responsive neural stimulation, temporal lobe epilepsy, rat

## Abstract

Responsive neural stimulation (RNS) is considered a promising neural modulation therapy for refractory epilepsy. Combined stimulation on different targets may hold great promise for improving the efficacy of seizure control since neural activity changed dynamically within associated brain targets in the epileptic network. Three major issues need to be further explored to achieve better efficacy of combined stimulation: (1) which nodes within the epileptogenic network should be chosen as stimulation targets? (2) What stimulus frequency should be delivered to different targets? and (3) Could the efficacy of RNS for seizure control be optimized by combined different stimulation targets together? In our current study, Granger causality (GC) method was applied to analyze epileptogenic networks for finding key targets of RNS. Single target stimulation (100 μA amplitude, 300 μs pulse width, 5s duration, biphasic, charge-balanced) with high frequency (130 Hz, HFS) or low frequency (5 Hz, LFS) was firstly delivered by our lab designed RNS systems to CA3, CA1, subiculum (SUB) of hippocampi, and anterior nucleus of thalamus (ANT). The efficacy of combined stimulation with different groups of frequencies was finally assessed to find out better combined key targets with optimal stimulus frequency. Our results showed that stimulation individually delivered to SUB and CA1 could shorten the average duration of seizures. Different stimulation frequencies impacted the efficacy of seizure control, as HFS delivered to CA1 and LFS delivered to SUB, respectively, were more effective for shortening the average duration of electrographic seizure in Sprague-Dawley rats (*n* = 3). Moreover, the synchronous stimulation of HFS in CA1 combined with LFS in SUB reduced the duration of discharge significantly in rats (*n* = 6). The combination of responsive stimulation at different targets may be an inspiration to optimize stimulation therapy for epilepsy.

## Introduction

Neural modulation is gradually accepted by those patients with medicine-refractory epilepsy who are not candidates for surgery resection (1, 2). Compared with Vagus nerve stimulation (VNS) and deep brain stimulation, which deliver scheduled stimulation on open-loop mode, responsive neural stimulation (RNS) delivers electrical stimulation in response to the neural activity of the target tissue and is emerging as one of the most promising approaches for refractory epilepsy treatment (3). Several clinical multi-center outcome studies demonstrated a median reduction in seizure frequency of 53% at 2 years and 72% at 6 years with the treatment of RNS (4, 5). However, the efficacy of RNS is still far from optimal due to the various stimulation parameters, complex stimulation targets for seizure control, and limited understanding of neural modulation mechanism (6). Stimulation targets and parameters of RNS are intimately related to the efficacy of seizure control. Understanding the knowledge of seizure initiation and propagation is crucial for looking for ideal stimulation targets. It has been demonstrated that limbic structures, primarily the hippocampus, amygdala, subiculum, and entorhinal cortex, are the sites of seizure initiation in temporal lobe epilepsy (TLE), moreover, the initiation and propagation varies between subjects (7). The targets for RNS are typically seizure onset zones in clinical studies. To optimize the efficacy, extensive studies were performed to look for more effective stimulation brain targets in recent studies (8–10). Most of the explored potential targets were those directly involved in seizure generation, propagation, or served as a hub to control an epileptic network (3). Specifically, besides the classical epileptic foci of CA3 and CA1 in hippocampi, other targets, such as subiculum (SUB) and anterior nucleus of thalamus (ANT) which have a tight connection with the mesial temporal lobe structure, were also proven to be potential stimulation targets of seizure suppression (11). Except for the stimulation targets, optimal stimulation parameters for aborting seizure activity are still unclear (12). Among the complex stimulus parameter space, stimulation frequency is a vital parameter and has distinct effects on different brain targets (13). In general, stimulation with a high frequency range (>70 Hz) is deemed to be effective for seizure control, since it may demonstrate the acute suppressive effects on neuronal synchrony by preferential activation of GABA-ergic inhibitory neurons and alter extracellular potassium concentrations (14). Nevertheless, stimulation with low frequency (<10 Hz) delivered to white matter tracts evokes a large, coordinated population burst which then leads to a period of reduced population firing mediated by slow after-hyperpolarization and GABA-B currents (15, 16), and attenuates seizure severity in multiple rodent models and non-human primate models (17, 18). Both high frequency and low frequency stimulations applied in animal and clinical studies were proven to be effective for seizure control (19–22). However, to the best of our knowledge, whether high frequency or low frequency stimulation applied at each different target has different efficacy for seizure control in the same animal model of temporal lobe epilepsy has not been well-explored. Overall, the identification of new targets and approaches for brain stimulation in epilepsy control is particularly compelling.

As the research progressed, epilepsy came to be understood as a disorder of the large neural network, the activity of which depends on the dysfunction of widespread regions in the brain rather than a single epileptic focus (23). Right now, the main approach of neural modulation for seizure control is delivered to a single target region alone, which may not sufficiently alter the dynamics of networks during seizures and may underline the suboptimal efficacy of RNS for seizure control (24). Therefore, it might be more effective to alter the dynamics of brain networks during seizures if responsive stimulation could be delivered to multi-targeted brain regions. Hence, simultaneously combined stimulations on different targets hold great feasibility for improving the efficacy of seizure control. Only a few studies were performed to evaluate this hypothesis. One of the most intriguing works was simultaneously activating inhibitory luminopsins on dentate gyrus and ANT of the rat brain, which was proven to be more effective than inhibition of each single individual structure (25). Overall, three major issues are worthy of further exploration to optimize the efficacy of RNS for seizure suppression: How to find key targets of combined stimulations in the epileptic network? How to choose the stimulus frequency of combined stimulation? And whether the efficacy could be optimized by combined stimulation matched with the ideal stimulus frequency of each different key target?

To answer the questions above, epileptic activity should be assessed in terms of functional connectivity and dynamics of neuronal networks (23). Among the many methods of functional connectivity, Granger causality (GC) is a reliable tool to estimate the interactions from time-series data during seizure onset and propagation (26, 27) and has the potential to help localize the ictal network (28, 29). In addition, if multiple neuronal groups have been recorded simultaneously, the conditional GC can distinguish the interaction relationship between direct vs. indirect interactions (30). In this work, epileptic activity was assessed in terms of functional connectivity and dynamics of neuronal GC method to find out key targets for brain stimulation. We then applied high frequency or low frequency responsive stimulation with our own designed RNS system for seizure control on a TLE rat model to evaluate the effect of single brain target stimulation on different potential targets for seizure control. Finally, combined stimulations with different groups of frequencies were delivered to key targets simultaneously to explore whether the combined stimulation with matching frequency could abort seizures efficiently.

## Materials and Methods

### Subject and Surgery

**Figure 1:**
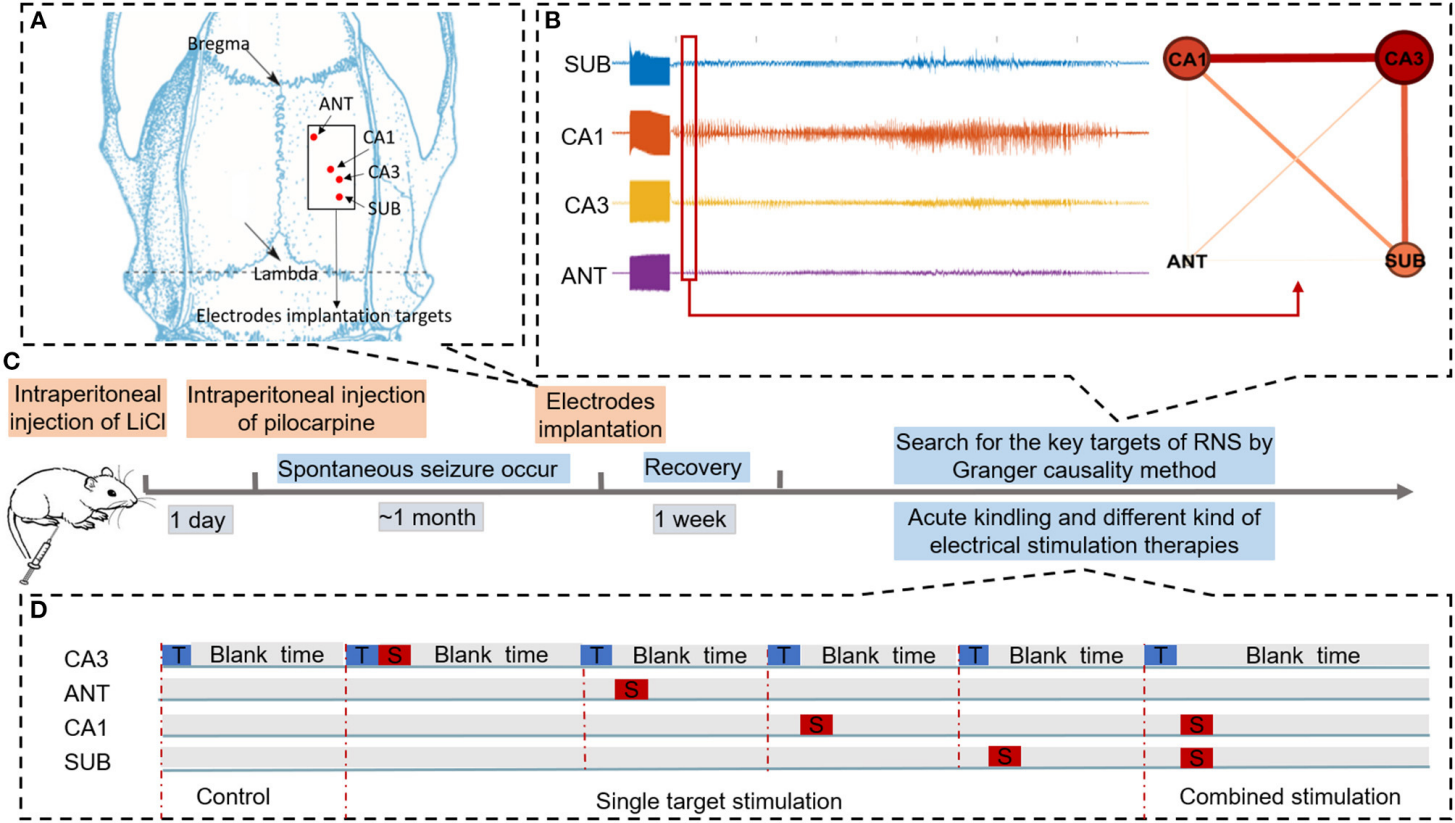
Schematic of experiments. **(A)** Schematic diagram of brain targets for electrode implantation. **(B)** Searching for key nodes of RNS during acute seizure onset within the epileptogenic network by Granger causality method. **(C)** Time flow chart of the experiment. **(D)** Three different conditions to evaluate the efficacy of different neural stimulation protocols for alleviating seizure severity. “T” in the blue rectangle represents trigger stimulation induced to R-CA3 to produce the acute seizure. “S” in the red rectangle represents therapeutic stimulation. In the control condition, there is only trigger stimulation delivered to CA3. In the single target stimulation condition, therapeutic stimulation was delivered to each of the four nodes. In combined stimulation, different therapeutic stimulation was delivered to both CA1 and SUB.

Sprague-Dawley rats weighing 250–300 g were used for induction of the chronic TLE model. The process of rats treated with lithium and pilocarpine for seizure induction was based on our prior work (31). In brief, a dose of lithium chloride (12.7 mg/100 g) was pre-administered to the rats intraperitoneally. One day later, atropine sulfate was injected into pretreated rats to reduce saliva secretion (1 ml, i.p., 30 min before pilocarpine injection). Rats were then treated with 32 mg/kg pilocarpine dissolved in saline and supplemented every 30 min by a repeated dose (16 mg/kg) until a sequence of animal behaviors of status epilepticus (SE) were observed. Once rats had SE lasting over 90 min, a dose of diazepam (20 mg/kg) was given to terminate the continuous convulsive seizure. After completing the whole process, rats were taken back to home cages and spontaneous seizures were monitored by video cameras. Approximately 1 month later, once spontaneous recurrent seizures were observed, rats were operated under propofol anesthesia and chronically implanted with tripolar electrodes for local field potentials' (LFPs) recording and neural stimulation. The tripolar electrode was made of 65 μm diameter teflo-coated microfilament electrodes, and their tips were spaced 500 μm from each other. One of the three electrodes was used for signal recording, and the other two strands (0.5 mm tip exposed) twisted together were paired for neural stimulation. Each rat was implanted with four tripolar electrodes for recording and stimulation, which were implanted into the ANT (−1.5 mm AP, +1.5 mm ML, −5.6 mmDV), CA1 (−3.6 mm AP, +2.0 mm ML, −3.0 DV) as shown in Figure 1A, CA3 (−4.2 mm AP, +3.0 mm ML, −3.7 mm DV), and subiculum (SUB, −6.0 mm AP, +3.0 mm ML, −3.0 mm DV) regions in the right hemisphere. In addition, a ground sliver electrode was implanted over the posterior fontanelle and a reference electrode was epidurally inserted on the region far from other record electrodes. After implantation, the electrodes were connected to a miniature receptacle. The whole assembly was finally fixed to the skull by dental cement. The whole process of our experiment was shown in Figure 1C. All experimental procedures were performed in accordance with the Animal Care and Use Committee of Zhejiang University and achieved ethical approval (Zhejiang University 15896).

### Acute Seizure Induction and Electroencephalography Acquisition and Analysis

Rats' behaviors were monitored by cameras and LFPs were recorded by our custom-made responsive neural stimulator; the detailed designs were described in our previous work (32–34) and depicted in Supplementary Figure 1. Specifically, an interface programmed on the computer was used in an online pattern, which contains neural signal recording function, real-time neural signal display interface, auto seizure detection function based on Ostu's algorithm (35), and real-time neural stimulation function to satisfy all needs of experiments in this work. The LFPs signals were recorded and digitized at 1,000 Hz, bandpass filtered from 0.1 to 500 Hz, notch filtered 50 Hz signal to reduce power frequency interference, and then shown in the display interface for seizure detection and stored for offline analysis.

The acute seizure induction experiment was performed 1 week after surgery. The process was similar to Tiwalade's prior work (36). Trigger stimulation, a sequence of biphasic square pulses with 200 μA amplitude, 1 ms pulse width, and 60 Hz frequency lasting for 5 s, was manually delivered to CA3 in the hippocampus to evoke acute seizures. The acute seizure onset was identified when the value of time-domain features including line-length (LL), average amplitude (AMP), and slope (SLP) all exceeded the threshold which was calculated by OSTU algorithm in all detected channels (34). Each acute seizure induction stimulation was performed with an interval longer than 10 min. Only those rats with six successive successful induction seizures were used as the subjects in the subsequent experiments.

### Key Targets Identification for Responsive Stimulation by Granger Causality Analysis

GC analysis was adopted to identify directed interaction between pairs of signals in multichannel signals. The main idea of GC is based on autoregressive (AR) modeling, in which a signal Y “Granger-causal” another signal X if the future of X can be predicted by the past Y and X. Moreover, the prediction error of X can be decreased with past Y as compared to only being predicted by the past values of X itself (37). For example, assuming two signals X(t) and Y(t) are covariance stationarity:

(1) $$\begin{array}{l} \begin{array}{c} X\left(t\right)=\sum_{j=1}^{p}A_{X}X\left(t-j\right)+\;\varepsilon_{1}\left(t\right) \end{array} \end{array}$$

(2) $$\begin{array}{l} \begin{array}{c} X\left(t\right)=\sum_{j=1}^{p}B_{XX}X\left(t-j\right)+\sum_{j=1}^{p}B_{XY}Y\left(t-j\right)+\varepsilon_{2}\left(t\right) \end{array} \end{array}$$

A univariate AR model of signal X is made by using past X values to predict future X values, as shown in (1). A bivariate AR model is calculated in (2), which combined the past X and Y to predict the future values of X. Here, p past values are included to predict the current value (p is the optimal AR model order), *A*_*X*_, *B*_*XX*_, *and B*_*XY*_ are the coefficients of AR models, and ε_1_, *andε*_2_ are the prediction errors of signal X, respectively. As shown in (3), *F*_*Y* → *X*_ is the interaction magnitude of GC (from Y to X), calculated by the log ratio of the prediction error variances for AR model. When Y reduces the prediction error of X, the log ratio is positive and Y “Granger-cause” X. Instead, the opposite interaction can be assessed by reversing the positions of two signals.

(3) $$\begin{array}{l} F_{Y\to X}=In\frac{var\left(\varepsilon_{1}\left(t\right)\right)}{var\left(\varepsilon_{2}\left(t\right)\right)} \end{array}$$

In this study, LFP data recorded from each of the four electrodes were analyzed for G-causality in the time domain using the Multivariate Granger Causality (MVGC) toolbox (38). GC was calculated in the first 5 s of seizure onset using the following parameters: window size of 2,000 samples, 50% window overlap, the number of surrogates 10 to determine the statistically significant connectivity between electrodes, and the AR model order was estimated using Bayesian Information Criterion (BIC) to a maximum of 30 lags, subsequently, pairwise GC values were calculated by MVGC routines (tsdata_to_var). then, unit causal density, cd_u_(i), which is the summed causal interactions involving node i, normalized by the number of nodes were measured for inferring the centricity of each electrode targeted in ANT, CA1, CA3, and SUB. Nodes with high values of cd_u_ can be considered to be causal hubs within the circuit (39). while unit causal density varied in order of magnitude from seizure to seizure and weighted cdu(i) [wcdu(i)] were calculated by cdu(i) dividing causal density [the sum of cdu(i)] to raise the comparability of data in different seizure event. The formula is depicted as follows, where n denotes the number of nodes.

(4) $$\begin{array}{l} cd_{u}\left(i\right)=\frac{1}{n}\underset{i\neq j}{\sum }F_{X_{i}\to X_{j}} \end{array}$$

(5) $$\begin{array}{l} wcd\left(i\right)=\frac{cd_{u}\left(i\right)}{\sum_{1}^{n}cd_{u}\left(i\right)} \end{array}$$

In order to illustrate the network by graphs clearly, networks were visualized by simple graphical depictions (Figure 1B); in this network, nodes represent targets of recording channels and edges represent G-causality between two nodes.

Generally speaking, cdu or wcdu of each node was a useful description of dynamic complexity, which reflected the total degree of inflow and outflow causal information. As is known, during the propagation of epileptic activity, transient information flow occurs among extensive brain networks. The brain regions with high cd_u_ or wcd_u_ values may indicate that they take an active part in the information interactions within the network. Therefore, these brain regions are considered to be causal hubs and are more likely targets than others for neuromodulation. Moreover, these nodes were likely to be key targets of RNS for seizure control.

### Single Target Stimulation on Key Targets

To evaluate the efficacy of seizure suppression in key targets which were found by GC, biphasic and charge-balanced therapeutic stimulation (100 μA amplitude, 300 μs pulse width, 5 s duration) with high frequency (130 Hz) or low frequency (5 Hz) was delivered to each of the four selected targets, namely SUB, CA3, CA1, and ANT, in three subjects after evoked seizures. As shown in Figure 1D, each rat will receive a single target stimulation condition and sham control condition, respectively, after acute seizure induction. The sham control condition received a fake stimulation without current after an evoked seizure was detected. While in the single target stimulation condition, therapeutic stimulation was delivered to one of the selected targets once acute seizure was detected. Each sham control condition trial was interleaved with one single target stimulation trial to access the efficacy of seizure control with different therapeutic protocols (high frequency or low frequency). To ensure the background of each trial is consistent, a 10-min interval was set between evoked stimulation delivery. Different stimulus types were randomly organized. For further statistical analyses, at least six trials were included in each different condition with different therapeutic stimulation protocols in the same test subject.

### Combined Stimulations of Key Targets

To explore whether the combined stimulations of key targets would improve the efficacy of seizure control, combined therapeutic stimulations were delivered to potential therapeutic targets evaluated by the single target stimulation experiment. The process of the combined stimulation experiment was similar to the single target stimulation experiment. The combined stimulation condition would deliver the different combinations of high and low frequency stimulations to different targets simultaneously.

### The Indicator of Seizure Severity

The duration of evoked seizures was counted as the indicator of seizure severity in this study since it was related to essential improvements in GABA functions (40). Teager energy (TE), which was found to be a reliable indicator for providing temporal markers for seizure onset and offset, was calculated in CA3 for counting seizure duration (41, 42). The LFP recorded in CA3 with 90 s before evoked stimulation was used as baseline. The criteria to select valid seizure activities was identified with a threshold of the mean of TE plus five times the standard deviation calculated by the baseline. Two typical examples of seizure duration counting were shown in Supplementary Figure 2.

### Statistics Methods

Two different statistical analysis methods were used in this work. Kruskal-Wallis was applied to assess the significance of wcd_u_ between each different node. Student's *t*-test (*t*-test) was used to evaluate the significance of electrographic seizure duration changes between the control condition and stimulation condition under different therapeutic stimulation protocols. A *p* < 0.05 was identified as statistically significant.

## Results

### Performance of Seizure Detection

The performance of the seizure detection algorithm was evaluated in six subjects before it was applied to our custom-made neural stimulator. As shown in Table 1, the seizure detection rate was 100% with an average 0.377 second time delay on account of a tiny step of the time window (120 ms) which was used for feature construction. However, a high false alarm rate was also reached in the meantime for the same reason. To reduce the impact of the false alarm, the function of auto seizure detection was disenabled until trigger stimulation for evoking acute seizure was delivered. Besides, once seizure onset was detected by the algorithm, the function of seizure detection was disenabled to ensure only one sequence of therapeutic stimulation was delivered for the corresponding evoked seizure treatment.

**Table 1:** The performance of seizure detection algorithm in the acute seizures.

**Subjects**	**Detected seizures**	**Detected duration(min)**	**Time Delay(s)**	**False alarm (/hr)**	**Detection rate**
#S1	17	85	0.035	8	100%
#S2	15	75	1.088	10	100%
#S*3*	17	85	0.113	2	100%
#S4	18	90	0.807	2	100%
#S5	15	75	0.048	4	100%
#S*6*	18	90	0.173	13	100%
Total	100	500	0.377	6.5	100%

### Key Targets Identification in The Temporal Lobe Epilepsy Model

**Figure 2:**
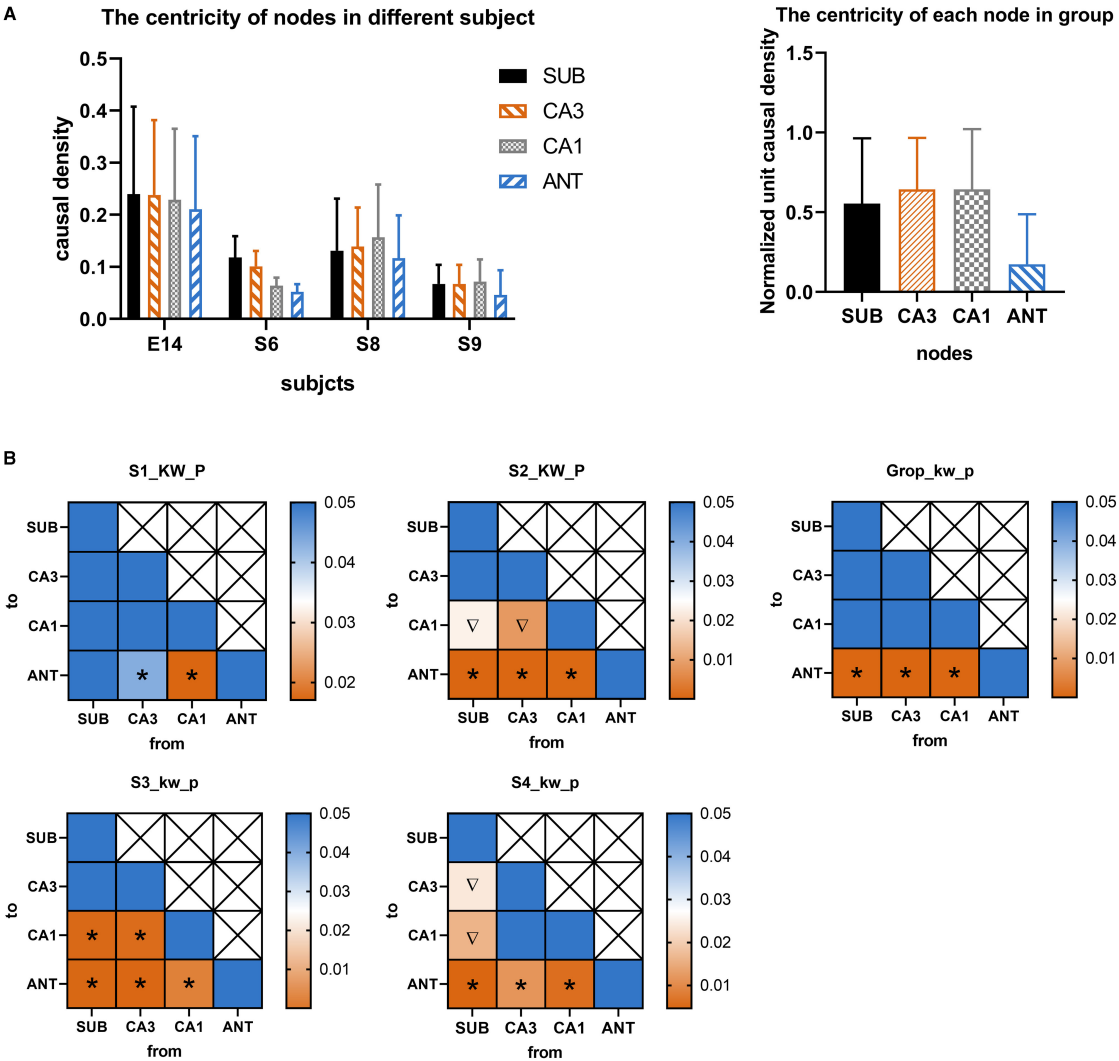
**(A)** The normalized weighted causality density (wcd_u_) of each node during seizure onset activity in different four rats. **(B)** Color-coded significance value matrices were obtained from the pairwise computation of wcd_u_ between each different node. The square with icon represents significant differences between two corresponding nodes. colors indicate the magnitude of the *p*-value. To outline the *p*-value small than 0.05, only *p*-value range from 0 to 0.05 were presented by colors. The symbol “*” represents wcd_u_ in the abscissa node is significantly larger than that in the ordinate node, while the symbol “∇” represents wcd_u_ in the abscissa node is significantly less than that in the ordinate node.

According to the GC analysis results, nodes with a high value of wcd_u_ were considered as key targets of RNS for aborting seizures at the seizure onset network. As shown in Figure 2, four rats with successful induction of seizures were used in this analysis. A total of 76 seizures, excluding those with motion artifacts, S1 (*n* = 18), S2 (*n* = 19), S3 (*n* = 20), and S4 (*n* = 19), were randomly selected from four subjects and used for G-causality analysis. The wcd_u_ depicted by Figure 2A indicated the importance of each corresponding node. Based on this result, the significance value of wcd_u_ between each two different nodes was calculated by Kruskal-Wallis to demonstrate the significant difference of importance degree (Figure 2B). The results indicated that the target with the highest value wcd_u_ was varied among different subjects, however, the target with the lowest value was ANT which is uniform among the four subjects.

**Figure 3:**
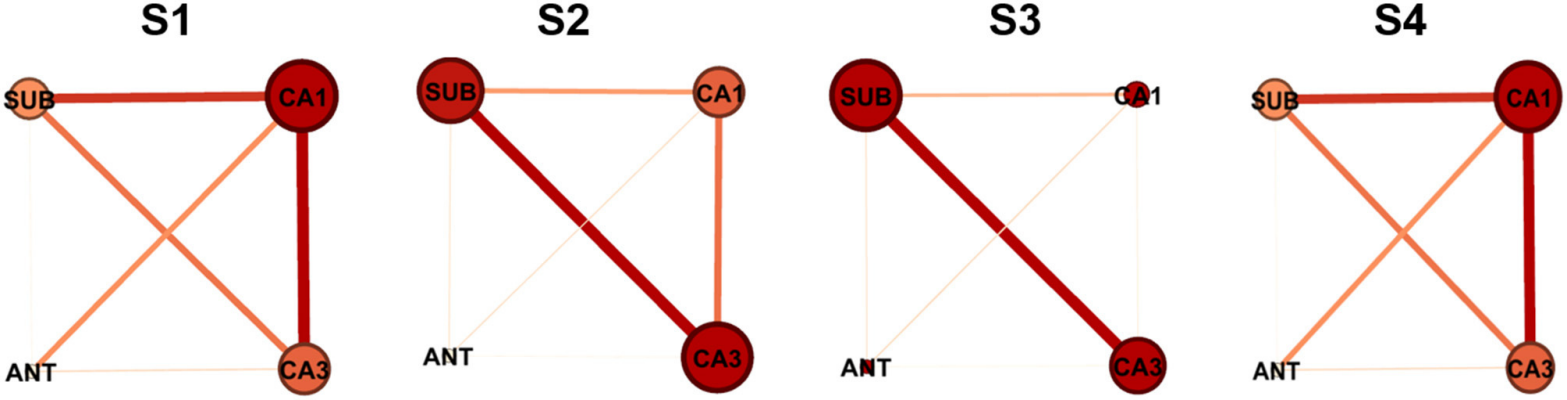
Network characteristics of the nodes in different four rats. The size of the node and the darkness of color are in direct proportion to the G-causality value between different nodes. The width of the line represents the interaction strength between pairs of nodes. In each network illustration, the line thickness of weighted edges is proportional to the GC values, when significantly. Node size and color are proportional to the Ncd_u_ value for each node.

To visualize the interactions between nodes, graphical networks of acute seizure onset were shown in Figure 3. The nodes represent different electrode targets and the width of the line represents the mean value of G-causality calculated above. The node with a larger size and a darker color is more likely to be the key target. In each rat individually, the key nodes found by graphical networks and interaction characteristics had a strong similarity between the results depicted by wcd_u_ in Figure 2 to some extent.

Above all, ANT should be excluded from key targets because it had the least influence among all stimulation targets. The target of CA1, CA3, and SUB played more important roles in acute seizures of our TLE rat model, which were identified as two key targets in the following study.

### Seizure Depression With Single Target Stimulation

**Figure 4:**
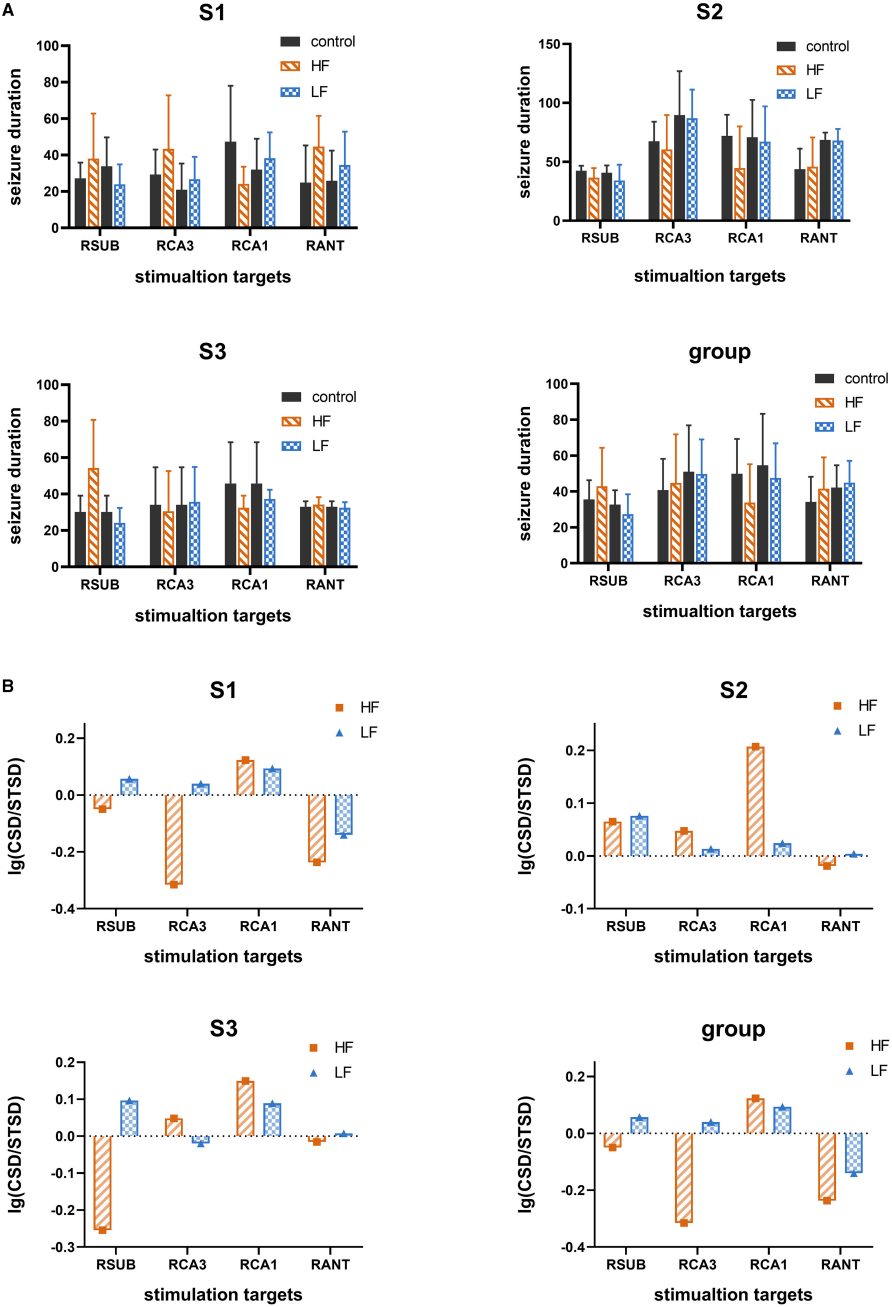
The therapeutic effect of single target stimulation for seizure suppression. **(A)** The seizure duration under control condition and single target stimulation condition with high frequency (HF, 130 Hz) or low frequency (LF, 5 Hz). Data are represented as mean ± standard error (M ± SEM). **(B)** Logarithm (to the base 10) of the average seizure duration's ratio. The ratio of average seizure duration in the control condition without therapeutic stimulation to that in single target therapeutic stimulation with high frequency (130 Hz) or with low frequency (5 Hz). The bar value higher than zeros represents it was effective for seizure suppression, the higher the better. “CSD” represent the average seizure duration under control condition which without therapeutic stimulation. “STSD” represents the average seizure duration under single target therapeutic stimulation.

To further evaluate the electrical stimulation efficacy on selected targets, high frequency stimulation (HFS, 130 Hz, 300 μs, 100 μA, 5 s) or low frequency stimulation (LFS, 5 Hz, 300 μs, 100 μA, 5 s) was delivered to one of four selected targets, namely SUB, CA3, CA1, and ANT, in three subjects. A total of 256 evoked seizures were statistically analyzed for evaluating the efficacy of single target stimulation. As shown in Figure 4A, there was no significant decrease (*t*-test, *p* > 0.05) of electrographic seizure duration between control condition and single target stimulation condition. The single target stimulation in all targets referred above was unable to shorten the electrographic seizure duration in this acute TLE model statistically. However, compared with other stimulation protocols, the result that LFS in SUB and HFS in CA1 reduced the average electrographic seizure duration was seen in all three rats (without a statistical difference). To further explore whether the HFS and LFS had different efficacy of seizure suppression, logarithm (to the base 10) of the ratio between average electrographic seizure duration in the control condition and that in stimulation condition was calculated. The average electrographic seizure duration in the control condition is longer than that in the stimulation condition when the logarithm value is bigger than zero. As displayed in Figure 4B, the results were consistent among three subjects that the logarithm value of LFS delivered to SUB is higher than HFS, while the logarithm value is larger when HFS was induced to CA1. Further, it also showed the stimulation protocol could shorten electrographic seizure duration. The larger value it has, the more effective it is. Therefore, it was suggested that SUB and CA1 may have a different response to LFS and HFS, while neither HFS and LFS could shorten the average seizure duration when delivered to ANT. In general, each different key node may have a distinct optimal stimulus frequency for seizure control. In this experiment SUB and CA1 may play a more important part than ANT for seizure control.

### Seizure Depression With Combined Stimulations

**Figure 5:**
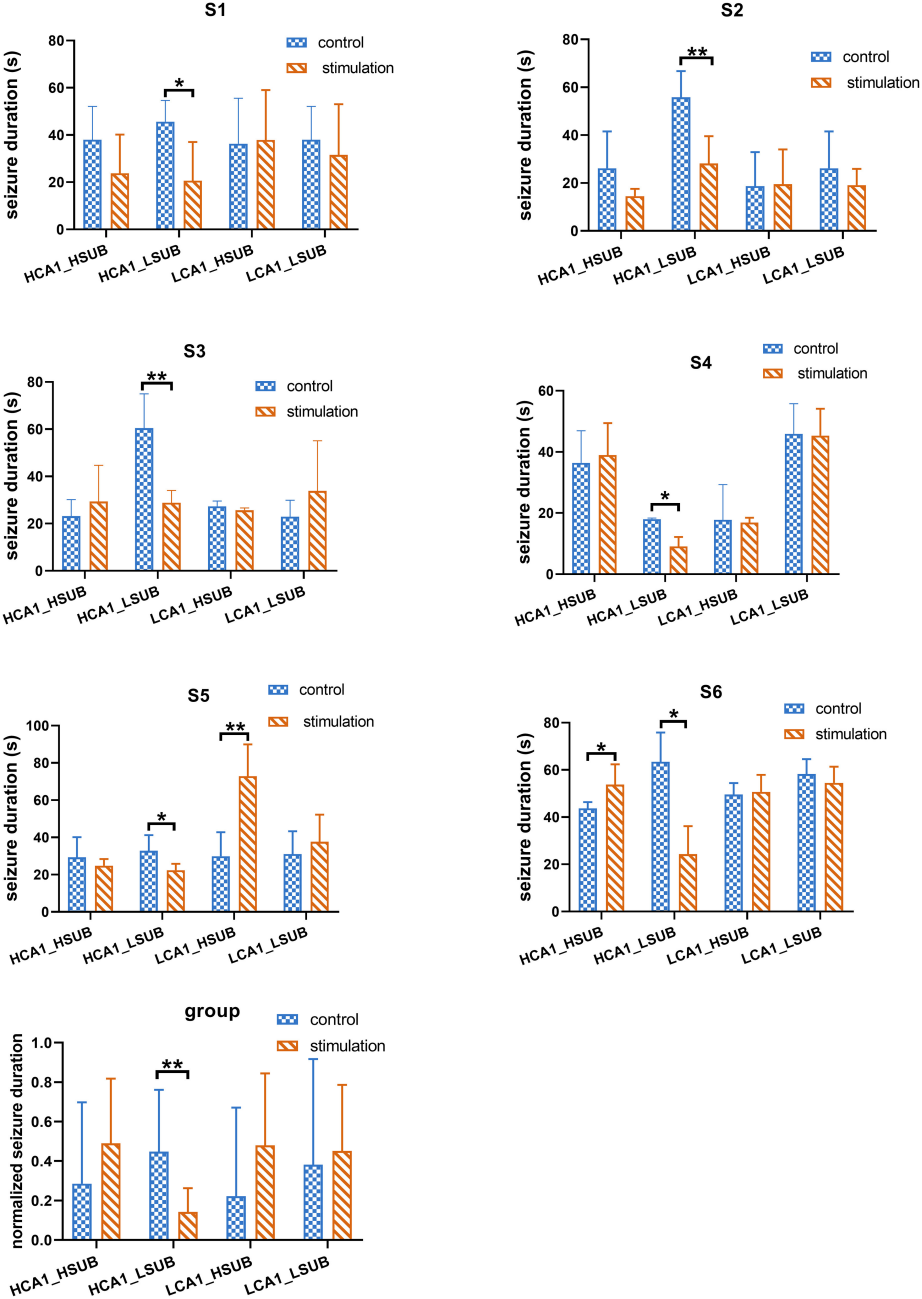
The efficacy of combined stimulation under different stimulation protocols. HF_HF represents that both CA1 and SUB received HFS simultaneously. HF_LF represents that CA1 received HFS while SUB received LFS. LF_HF represents that LFS delivered to CA1 while HFS delivered to SUB. LF_LF represents LFS delivered to both CA1 and SUB in the same time. “*” represents *p* < 0.05. “**” represents *p* < 0.01.

Since the single target stimulation approach may underline the suboptimal efficacy of seizure control, it is important to consider whether the efficacy could be improved by combined stimulation. We further tested the combined stimulation on SUB and CA1 with different stimulation frequencies. Another three subjects were included in the combination stimulation experiment, as shown in Figure 5, the duration of 288 evoked seizures under combined stimulation in total six different subjects were statistically analyzed. The results illustrated only the combination of HFS in CA1 and LFS in SUB could significantly decrease the electrographic seizure duration of evoked seizures in all six tested subjects (*t*-test, *p* < 0.05). This result matched with the above single target stimulation result, that combined stimulations were able to shorten electrographic seizure duration efficiently when the stimulation frequency matched with optimal frequency in each target. It is particularly important to point out stimulation with unmatched frequency might even enhance the seizure activity, which may worsen the treatment of epilepsy. As shown in Figure 5, combined HFS in SUB with LFS in CA1 significantly increased the electrographic seizure duration in rat S5. Similarly, the seizure severity also deteriorated when both CA1 and SUB received HFS in rat S6.

## Discussion

The purpose of this research was to investigate whether the efficacy of brain stimulation for seizure control could be improved by combined stimulations of key targets with matched frequency. Three main findings could be noted as follows: (1) Key targets were proven more effective in seizure control tested by single target stimulation experiment, which was in line with the targets found by GC method; (2) The stimulus frequency played an important role in the stimulation approach for seizure control. Each different key node may have a prior stimulus frequency between 130 and 5 Hz for seizure control since LFS delivered to SUB and HFS delivered to CA1 could shorten the average electrographic seizure duration; and (3) Combined stimulation with matched frequency could significantly decrease the duration of evoked electrographic seizures, which was more effective than single target stimulation.

Epilepsy is a network disorder with potential aberrance in nodes and/or pathways (43). A deeper understanding of the dynamics of epileptogenic networks may control and regulate seizure activity more effectively. As a way to measure the dynamic of epileptogenic networks, GC estimates of connectivity in the network have been shown to have some reference value. It has shown similar results to dynamic causal modeling, which has plausible estimates of human seizure propagation pathway and is in line with pathways demonstrated with DTI as well (44). However, the mathematical protocol for epileptogenic network analysis like GC does not merely help in understanding those progresses but also has a guiding value for establishing the RNS treatment strategies for epilepsy. Such techniques are practical and have potential to be used in clinical treatment. In this work, GC was used to find the key targets which have tight interconnections with other nodes during seizure onset, and it has the potential to aid therapeutic intervention like RNS. The single target stimulation for evaluating the efficacy of seizure suppression in each different node showed that the mean electrographic seizure duration could be shortened when key targets found by GC received matched therapeutic stimulation, while others are not. It suggested that GC could provide valuable insights into looking for potential targets for RNS in the specific epileptogenic network, though much more work should be carried out to support this conclusion.

The current neuromodulation technique is considered as a complementary rather than alternative treatment option to those patients who cannot benefit from conventional treatment (3). The approach of single target stimulation may underline the limited efficacy, since lots of regions involved in epileptic activity may occupy a vital position in seizure onset (45, 46). Therefore, the stimulation delivered to a single region alone might not abort seizure activity sufficiently (25, 46), which was also supported by a single target stimulation experiment in this study. To sum up, it is worthwhile to investigate the combined stimulation method (47). However, to our knowledge, only a few studies were performed on this aspect. Li et.al concentrated on a novel electrical stimulation approach involving distributed multielectrode microstimulation at the epileptic focus, which proved that distributed stimulation delivered together may be more effective to seizure control (48). Bertram et.al mainly focused on the circuits that support the different stages of seizures developed from a system's view of epilepsy. But this study was at the theoretical stage and no animal experiment was performed to evaluate the relationship between the epileptogenic network and stimulation pattern (46). Tung et.al introduced how precise multi-focal control of pathological circuits with optogenetic stimulation can be advantageous for the treatment of epilepsy (25). Nevertheless, whether a similar answer would be achieved on RNS, a more clinically achievable method has not been explored. In the current research, we only chose key targets that were found by GC method and evaluated by single target stimulation as our targets for combined stimulations. It is interesting to note that the key targets found by GC method are not exactly the most effective node for decreasing the average electrographic seizure duration in our acute TLE model. CA3 was proven to be a key node in seizure onset by GC but stimulation of CA3 was unable to shorten electrographic seizure duration in the single target stimulation experiment, which is not consistent with the previous studies (49). One possible explanation for the ineffective CA3 stimulation is that the trigger stimulation was also located in CA3 shortly before (3s in average) therapeutic stimulation. More studies of single or combined target brain stimulation could be performed on the chronic TLE model which may mimic clinical situations better.

In addition to the location of stimulation, the frequency of stimulation is another crucial factor of brain stimulation that inhibits seizure activity. Both HFS and LFS were studied for seizure control. HFS has been proven to be effective in many clinical and animal studies and LFS of a white matter tract reduced epileptiform discharges and seizures in patients (13). Similar to our result, HFS was more effective than LFS in CA1, which is in accord with a prior work that focused on comparing the efficacy between HFS and LFS delivered to the hippocampus in epileptic rats (50). Not all of our results were consistent with other studies. Both HFS and LFS delivered to SUB were demonstrated to be effective in seizure control (22, 51). However, LFS was superior to HFS in SUB in our current study. Moreover, the combined stimulation with matched frequencies in SUB and CA1 illustrated more effectiveness in seizure control. The effectiveness of combined stimulation may partly be on account of the stimulation energy since the combined stimulation will deliver twice as much electricity into the brain compared with single target stimulation. On the other hand, the matched optimal stimulation frequency is the most important factor for effective seizure control, for combined stimulation with unmatched frequency increased seizure duration conversely. These findings indicated that the efficacy of combined stimulation may be achieved by accumulating the effect of single target stimulation.

The evoked stimulation only induced to those pilocarpine model with spontaneous seizure; it demonstrated many pathophysiological mechanisms associated with epileptogenesis already exist before induced stimulation, including mossy fiber sprouting and interneuron loss ad granule cell dispersion in the dentate gyrus (52). It increased sensitivity for induced seizures to the kindling model in this pilocarpine-pretreated model. In our study, every induced electrographic seizure combined with behavior, however, it is a pity that not all behavior combined with electrographic seizure was collected and analyzed in this study. There was an essential difference between a spontaneous seizure and induced seizure since the stimulation targets located in CA3 may the reason CA1 and SUB have a higher GC value than ANT, although this model still provided a platform for us to study effective RNS protocols to reduce the severity of seizures. Effective RNS remains a choke point for long-term spontaneous seizure detection.

Our findings in this work provide valuable insights into the combined brain stimulation approach to improve the efficacy of seizure control. We do not deny the existence of other effective protocols of combined stimulation. However, it is difficult to evaluate the most effective one for seizure control by going through all the combined stimulation protocols, due to the existence of sophisticated conditions in this work. Besides, the stimulation frequencies selected in this current work were according to previous works and mainly depended on trial and error. Moreover, the acute TLE model was used in this study because of the limitation of the seizure detection algorithm, which has a very unified seizure type among different trials and subjects similar to kainic acid model (53). This is also different from various seizure onset types that existed in the clinical and chronic TLE animal model. If the different combinations of key targets did exist in different seizure onset types, it is still important to consider how to alter the approach of brain stimulation to improve the efficacy of seizure control. Combining all key targets found in different types of seizures together or a adaptive stimulation will be needed in such a situation. Therefore, more focus is needed on the intrinsic relationship between the epileptogenic network and stimulus parameters, and more methods are required to find out the most appropriate brain modulation method for refectory epilepsy in the future.

## Data Availability Statement

The original contributions presented in the study are included in the article Supplementary Material, further inquiries can be directed to the corresponding author/s.

## Ethics Statement

The animal study was reviewed and approved by Zhejiang University.

## Author Contributions

FZ, YZ, and KX designed this study. FZ organized the data. FZ and YY performed the data analysis and drafted the manuscript. JZ, PW, and KX revised the manuscript. All authors approved the final manuscript.

## Conflict of Interest

The authors declare that the research was conducted in the absence of any commercial or financial relationships that could be construed as a potential conflict of interest.

## Publisher's Note

All claims expressed in this article are solely those of the authors and do not necessarily represent those of their affiliated organizations, or those of the publisher, the editors and the reviewers. Any product that may be evaluated in this article, or claim that may be made by its manufacturer, is not guaranteed or endorsed by the publisher.
