# Supplementary material for: Combination of Matching Responsive Stimulations of Hippocampus and Subiculum for Effective Seizure Suppression in Temporal Lobe Epilepsy
Source: Front Neurol. 2021 Aug 26;12:638795. doi: 10.3389/fneur.2021.638795 (PMC8426572; doi:10.3389/fneur.2021.638795)
Supplement: Supplementary file 1 [file Data_Sheet_1.docx]

Supplementary Material


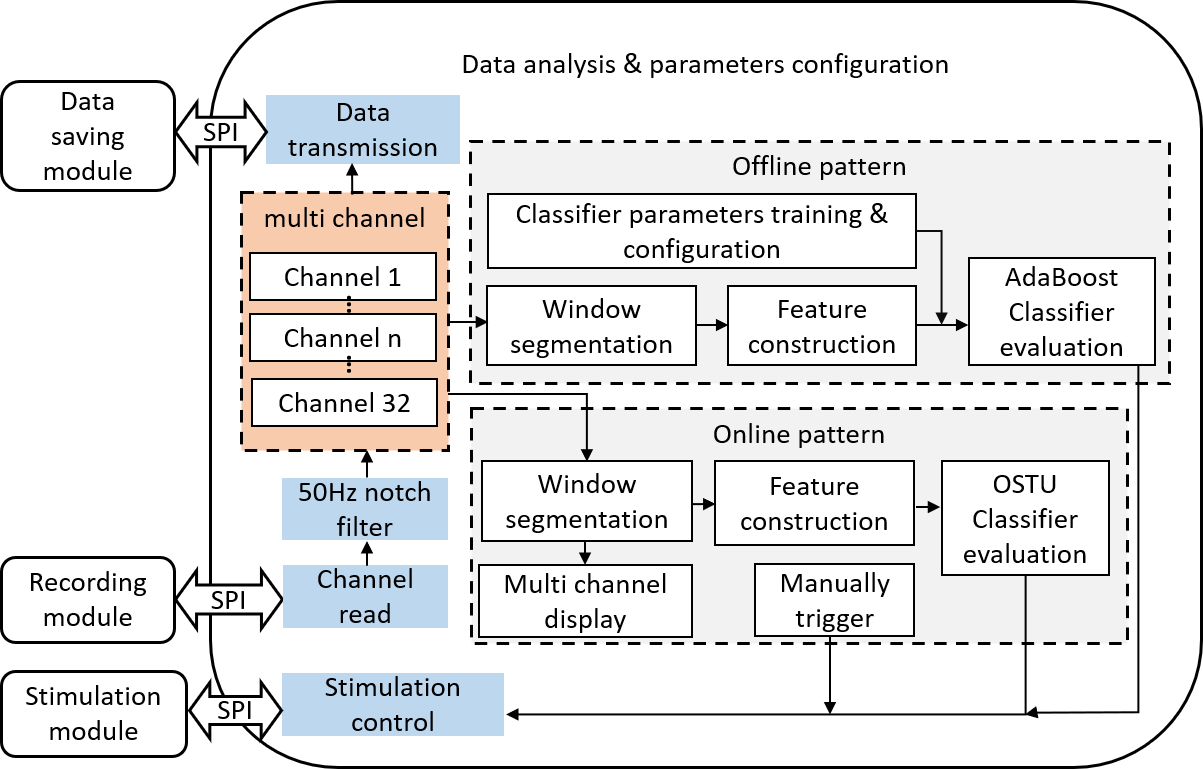


Figure S1. The architecture diagram of custom-made responsive neural stimulator.


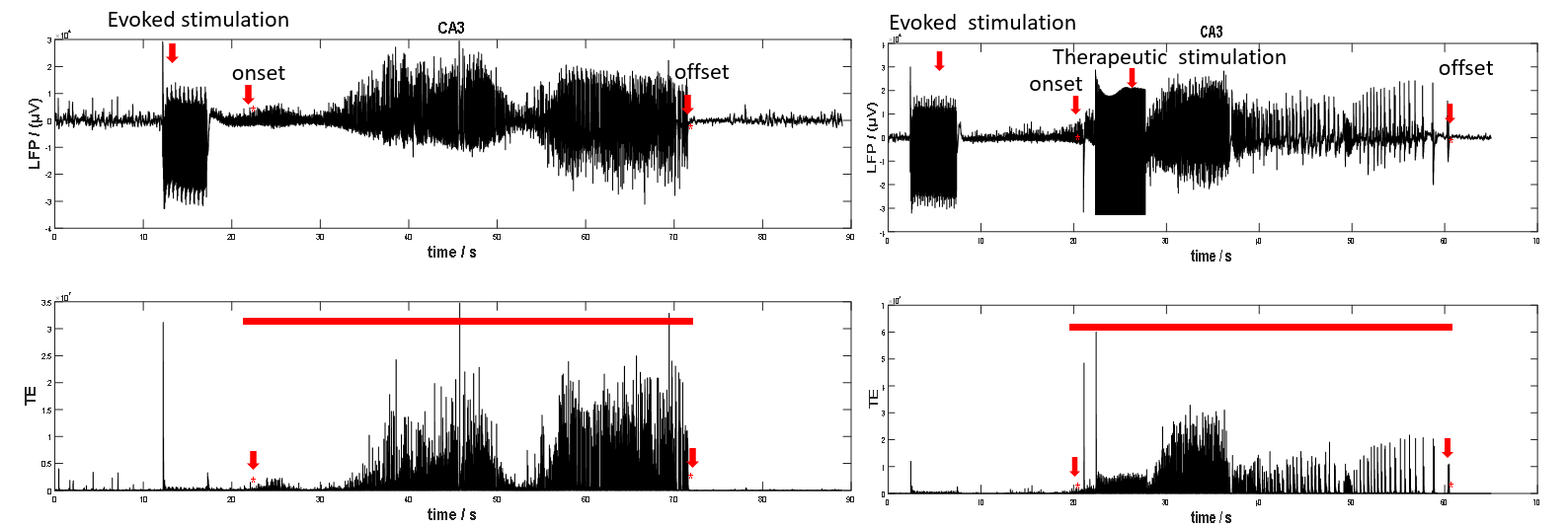


Figure S2. LFPs and corresponding Teager energy (TE) recorded in a rat from CA3 during evoked seizures with or without therapeutic stimulation. The red symbol ‘*’ represent the onset or offset of seizures. Red bars indicate period of seizure activities.
